# Supplementary material for: Multi‐dataset identification of innovative feature genes and molecular mechanisms in keratoconus
Source: J Cell Mol Med. 2024 Sep 19;28(18):e70079. doi: 10.1111/jcmm.70079 (PMC11412914; doi:10.1111/jcmm.70079)
Supplement: Supplementary file 2 — Material S2. [file JCMM-28-e70079-s002.zip › 7.GSEA analysis/2.ATOH7/KEGG_ARRHYTHMOGENIC_RIGHT_VENTRICULAR_CARDIOMYOPATHY_ARVC.html]

Details for gene set KEGG\_ARRHYTHMOGENIC\_RIGHT\_VENTRICULAR\_CARDIOMYOPATHY\_ARVC[GSEA]

|  || Dataset | ATOH7.group.cls#h\_versus\_l |
| Phenotype | group.cls#h\_versus\_l |
| Upregulated in class | h |
| GeneSet | KEGG\_ARRHYTHMOGENIC\_RIGHT\_VENTRICULAR\_CARDIOMYOPATHY\_ARVC |
| Enrichment Score (ES) | 0.4839457 |
| Normalized Enrichment Score (NES) | 1.5193421 |
| Nominal p-value | 0.032015067 |
| FDR q-value | 0.2752241 |
| FWER p-Value | 0.746 |
Table: GSEA Results Summary

  

Fig 1: Enrichment plot: KEGG\_ARRHYTHMOGENIC\_RIGHT\_VENTRICULAR\_CARDIOMYOPATHY\_ARVC      
 Profile of the Running ES Score & Positions of GeneSet Members on the Rank Ordered List

  

| SYMBOL | TITLE | RANK IN GENE LIST | RANK METRIC SCORE | RUNNING ES | CORE ENRICHMENT || 1 | CACNA2D1 | na | 19 | 0.977 | 0.0824 | Yes |
| 2 | LEF1 | na | 735 | 0.492 | 0.1035 | Yes |
| 3 | CACNA2D2 | na | 743 | 0.489 | 0.1448 | Yes |
| 4 | SLC8A1 | na | 1100 | 0.432 | 0.1712 | Yes |
| 5 | CDH2 | na | 1265 | 0.412 | 0.2014 | Yes |
| 6 | ITGA5 | na | 1306 | 0.407 | 0.2348 | Yes |
| 7 | CACNA2D4 | na | 1506 | 0.385 | 0.2618 | Yes |
| 8 | SGCB | na | 1611 | 0.376 | 0.2907 | Yes |
| 9 | CACNG3 | na | 2317 | 0.322 | 0.2976 | Yes |
| 10 | CACNG4 | na | 2923 | 0.284 | 0.3043 | Yes |
| 11 | ACTN2 | na | 2998 | 0.281 | 0.3260 | Yes |
| 12 | TCF7 | na | 3059 | 0.277 | 0.3478 | Yes |
| 13 | ITGB8 | na | 3379 | 0.261 | 0.3607 | Yes |
| 14 | CACNB4 | na | 3383 | 0.261 | 0.3828 | Yes |
| 15 | PKP2 | na | 4040 | 0.235 | 0.3838 | Yes |
| 16 | DSG2 | na | 4484 | 0.219 | 0.3895 | Yes |
| 17 | ITGA1 | na | 4519 | 0.218 | 0.4070 | Yes |
| 18 | ACTN1 | na | 4698 | 0.211 | 0.4198 | Yes |
| 19 | RYR2 | na | 4883 | 0.205 | 0.4319 | Yes |
| 20 | CACNG6 | na | 4974 | 0.202 | 0.4464 | Yes |
| 21 | ITGB3 | na | 5329 | 0.191 | 0.4524 | Yes |
| 22 | ACTN3 | na | 5743 | 0.178 | 0.4556 | Yes |
| 23 | SGCA | na | 5781 | 0.177 | 0.4696 | Yes |
| 24 | CACNB3 | na | 6009 | 0.171 | 0.4775 | Yes |
| 25 | TCF7L1 | na | 6644 | 0.153 | 0.4722 | Yes |
| 26 | ITGA6 | na | 6683 | 0.152 | 0.4839 | Yes |
| 27 | ITGA2 | na | 8085 | 0.117 | 0.4535 | No |
| 28 | CTNNA3 | na | 8333 | 0.112 | 0.4558 | No |
| 29 | CACNA1S | na | 8751 | 0.103 | 0.4525 | No |
| 30 | ITGA7 | na | 8847 | 0.102 | 0.4584 | No |
| 31 | ITGAV | na | 8959 | 0.100 | 0.4637 | No |
| 32 | ITGB1 | na | 9539 | 0.090 | 0.4546 | No |
| 33 | ITGB5 | na | 10348 | 0.076 | 0.4377 | No |
| 34 | ITGA3 | na | 10756 | 0.070 | 0.4319 | No |
| 35 | SGCD | na | 11879 | 0.054 | 0.4041 | No |
| 36 | CACNB2 | na | 12833 | 0.042 | 0.3802 | No |
| 37 | ITGA10 | na | 13121 | 0.039 | 0.3752 | No |
| 38 | GJA1 | na | 13928 | 0.031 | 0.3546 | No |
| 39 | JUP | na | 15727 | 0.015 | 0.3040 | No |
| 40 | ITGB4 | na | 15745 | 0.015 | 0.3048 | No |
| 41 | ITGA2B | na | 16498 | 0.009 | 0.2838 | No |
| 42 | ACTG1 | na | 16994 | 0.005 | 0.2699 | No |
| 43 | CTNNB1 | na | 18214 | -0.006 | 0.2352 | No |
| 44 | LMNA | na | 18712 | -0.010 | 0.2218 | No |
| 45 | CTNNA1 | na | 18820 | -0.011 | 0.2196 | No |
| 46 | ITGA8 | na | 19267 | -0.015 | 0.2081 | No |
| 47 | DES | na | 20139 | -0.023 | 0.1849 | No |
| 48 | ITGA11 | na | 20534 | -0.027 | 0.1758 | No |
| 49 | CACNG1 | na | 21206 | -0.034 | 0.1593 | No |
| 50 | ACTN4 | na | 21725 | -0.040 | 0.1477 | No |
| 51 | DSP | na | 21766 | -0.040 | 0.1500 | No |
| 52 | CACNA1C | na | 22079 | -0.044 | 0.1447 | No |
| 53 | TCF7L2 | na | 22134 | -0.044 | 0.1469 | No |
| 54 | CACNG5 | na | 22404 | -0.047 | 0.1432 | No |
| 55 | ACTB | na | 22412 | -0.047 | 0.1470 | No |
| 56 | CTNNA2 | na | 22741 | -0.051 | 0.1419 | No |
| 57 | DMD | na | 23011 | -0.055 | 0.1388 | No |
| 58 | ITGA9 | na | 23027 | -0.055 | 0.1430 | No |
| 59 | DSC2 | na | 23049 | -0.055 | 0.1471 | No |
| 60 | ATP2A2 | na | 23505 | -0.062 | 0.1393 | No |
| 61 | LAMA2 | na | 24087 | -0.071 | 0.1285 | No |
| 62 | ITGA4 | na | 26362 | -0.112 | 0.0724 | No |
| 63 | CACNA2D3 | na | 26367 | -0.112 | 0.0818 | No |
| 64 | CACNA1D | na | 26374 | -0.112 | 0.0911 | No |
| 65 | ITGB7 | na | 26886 | -0.123 | 0.0868 | No |
| 66 | CACNG2 | na | 26917 | -0.123 | 0.0964 | No |
| 67 | EMD | na | 26971 | -0.125 | 0.1055 | No |
| 68 | CACNA1F | na | 27070 | -0.127 | 0.1134 | No |
| 69 | ITGB6 | na | 27368 | -0.135 | 0.1163 | No |
| 70 | CACNG7 | na | 28246 | -0.158 | 0.1044 | No |
| 71 | DAG1 | na | 30263 | -0.220 | 0.0649 | No |
| 72 | SGCG | na | 30268 | -0.220 | 0.0834 | No |
| 73 | CACNB1 | na | 31307 | -0.259 | 0.0755 | No |
| 74 | CACNG8 | na | 31602 | -0.274 | 0.0902 | No |
Table: GSEA details [plain text format]

  

Fig 2: KEGG\_ARRHYTHMOGENIC\_RIGHT\_VENTRICULAR\_CARDIOMYOPATHY\_ARVC      
 Blue-Pink O' Gram in the Space of the Analyzed GeneSet

  

Fig 3: KEGG\_ARRHYTHMOGENIC\_RIGHT\_VENTRICULAR\_CARDIOMYOPATHY\_ARVC: Random ES distribution      
 Gene set null distribution of ES for **KEGG\_ARRHYTHMOGENIC\_RIGHT\_VENTRICULAR\_CARDIOMYOPATHY\_ARVC**
